# Whole-Genome Sequence of *Chlamydia abortus* Strain GN6 Isolated from Aborted Yak Fetus

**DOI:** 10.1128/genomeA.00893-17

**Published:** 2017-08-31

**Authors:** Zhaocai Li, Jinshan Cai, Xiaoan Cao, Zhongzi Lou, Yilin Chao, Wei Kan, Jizhang Zhou

**Affiliations:** a State Key Laboratory of Veterinary Etiological Biology, Lanzhou Veterinary Research Institute, Chinese Academy of Agricultural Sciences, Lanzhou, Gansu, People’s Republic of China; b Center for Animal Disease Control and Prevention in Qinghai Province, Xi'ning, Qinghai, People’s Republic of China

## Abstract

The obligate intracellular Gram-negative bacterium *Chlamydia abortus* is one of the causative agents of abortion and fetal loss in sheep, goats, and cattle in many countries. It also affects the reproductivity of yaks (*Bos grunniens*). This study reports the whole-genome sequence of *Chlamydia abortus* strain GN6, which was isolated from aborted yak fetus in Qinghai-Tibetan Plateau, China.

## GENOME ANNOUNCEMENT

*Chlamydia abortus* is one of the etiological agents of abortion in pregnant ruminants and some other mammals (1) and represents a zoonotic risk to humans (2, 3). The pathogen is distributed worldwide and has been shown to have relatively low genetic heterogeneity with six multilocus variable-number tandem-repeat analysis (MLVA) genotypes which, to a large extent, are related to their geographical origin (4, 5). Recently, yak flocks have demonstrated the severe problem of abortion associated with *Chlamydia* infection in several regions of the Qinghai-Tibetan Plateau, China (6). We have isolated several *Chlamydia* strains from the aborted yak fetuses and identified them as *C. abortus* belonging to the genotype 2 group (7). For controlling the disease, an inactivated vaccine was developed using one of the isolated strains, GN6, which exhibited high virulence. Administration of the vaccination significantly decreased the abortion rate in the yak flocks with a protective rate of over 95% (data not shown). Here we report the genome sequence of GN6, which will contribute to the understanding of the genetic diversity and antigen properties of *C. abortus*.

The *C. abortus* GN6 genome was sequenced using PacBio sequencing technology. A total of 174,311 reads were obtained, with an *N*_50_ read length of 10,815 bp and a mean length of 7,863 bp. Approximately 280× depth was achieved for the bacterial genome. *De novo* assembly was performed with the software program Canu (version 1.0) (8), and the genome sequences were assembled in one scaffold without any gaps. *C. abortus* GN6 is composed of a 1,144,357-bp circular chromosome with a G+C content of 39.86%. A total of 1,031 putative genes in the chromosome were predicted by use of the NCBI Prokaryotic Genome Annotation Pipeline, of which 987 were coding sequences and 44 were RNA genes. The genome contains 38 tRNAs, 3 noncoding RNAs (ncRNAs), and 3 types of rRNAs (5S, 16S, and 23S) in one rRNA operon. There are 62 predicted pseudogenes in the strain, which is more pseudogene content than in the reference strains S26/3 and LLG and indicates the genetic heterogeneity of this isolate (9, 10).

### Accession number(s).

This complete genome sequence shotgun project of *C. abortus* strain GN6 has been deposited at DDBJ/EMBL/GenBank under the accession no. CP021996, and the version cited in this paper is CP021996.1.
